# Supplementary material for: Predicting learning achievement using ensemble learning with result explanation
Source: PLoS One. 2025 Jan 2;20(1):e0312124. doi: 10.1371/journal.pone.0312124 (PMC11694977; doi:10.1371/journal.pone.0312124)
Supplement: Attachment — Submitted filename: Response to Reviewers.pdf [file pone.0312124.s001.pdf]

Original Manuscript ID: PONE-D-24-26784

Original Article Title: “Predicting Learning Achievement Using Ensemble Learning with Result Explanation”

Dear Editor,

Thank you for allowing a resubmission of our manuscript, with an opportunity to address the reviewers’ comments.

We are uploading (a) our point-by-point response to the comments (below) (response to reviewers), (b) an updated manuscript with yellow highlighting indicating changes. and (c) a clean updated manuscript without highlights (PDF main document).

We sincerely hope this manuscript will be accepted for publication on *PLOS ONE*.

Best regards,

<Tingting, Tong>et al.

Reviewer#1, Concern # 1: The authors should mention the main contributions and novelty at the end of the introduction section.

Author response: We appreciate your feedback on the importance of clearly highlighting the main contributions and novelty of our work in the introduction section.

In response to your suggestion, we have revised the introduction to clearly state the primary contributions and novel aspects of our research. Specifically, we have added a new paragraph at the end of the introduction, which is presented as follows:

> To summarize, the main contributions and novelty of our work are as follows:
>
> - We developed a robust ensemble learning model that integrates six distinct machine learning models (K-Nearest Neighbor, Naive Bayes, Random Forest, Gradient Boosting Decision Tree, eXtreme Gradient Boosting, and Multi-Layer Perceptron) as base learners, with Logistic Regression as the meta learner. This model effectively addresses the biases and limitations of previous methodologies.
> - To enhance the interpretability of our predictions, we employed the SHapley Additive exPlanation (SHAP) for feature importance analysis. This allowed us to identify critical factors influencing learning achievement, providing actionable insights for more precise and targeted interventions.
> - The effectiveness of the proposed model was verified through experiments on the XuetangX dataset. The proposed model outperforms traditional machine learning and deep learning methods in terms of prediction accuracy.
>
> The remainder of this paper is structured as follows: Section 2 reviews related work; Section 3 outlines the overall research framework and details the proposed model structure; Section 4 presents the experimental results and analysis; and Section 5 summarizes the conclusions and limitations of the study.

This paragraph concisely outlines the main contributions of our research, emphasizing their significance and novelty in the field. We believe that this addition enhances the clarity and impact of our introduction. Thank you once again for your valuable insights.

Reviewer#1, Concern # 2: The authors mentioned the stacking ensemble-based learning model but it's not properly explained. I suggest providing a section related to stack ensemble learning with its proper procedure.

Author response: We sincerely apologize for the unclear expression in the original manuscript. We appreciate your insightful observation regarding the need for a more comprehensive explanation of the stacking ensemble-based learning model. We recognize that providing a detailed description of this model and its procedure is essential for readers to fully understand the methodology and its implications.

In response to your suggestion, we have added a new section specifically dedicated to

explaining the stacking ensemble learning model. This section is presented as follows:

Originally introduced by David H. Wolpert in 1992 [48], stacking differs from Bagging and Boosting by combining the outputs of diverse base learners through a meta learner rather than using identical base models. This method, illustrated in Fig 5, enhances model robustness and flexibility, making it a powerful tool in ensemble learning. The process involves splitting the training set into k folds, training base learners on k-1 folds, and using these predictions to train the meta learner. For this study, k was set to 5. The implementation of a two-layer stacking framework involves the following four steps:

- Divide the Training Set: Split the training set into 5 folds using 5-fold cross-validation.
- Train Base Learners: Train the base learners on 4 folds and predict the 5-th fold. Repeat this process for each fold, then concatenate the predictions to form a new training set while retaining the original labels.
- Train Meta Learner: Train the meta learner on the new training set constructed from the base learners' predictions.
- Predict New Test Set: Use the trained meta learner to predict the new test set samples, yielding the final predictions.

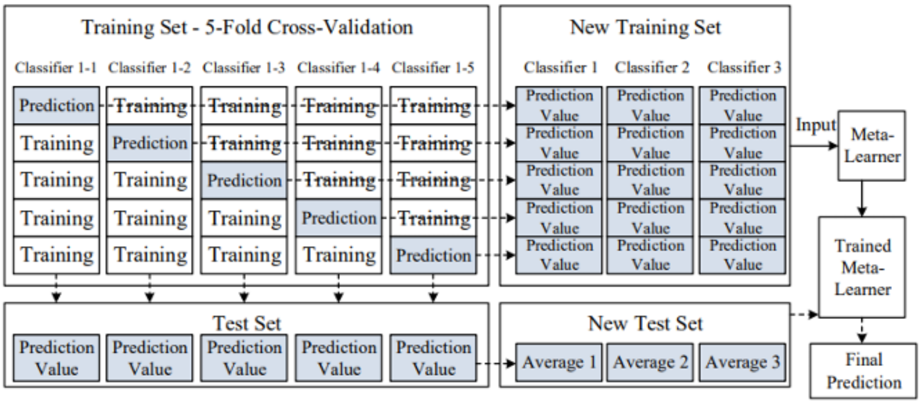

Fig 5. Flowchart of the Stacking method.

Reviewer#1, Concern # 3: For the reader concerns the authors should incorporate the recent predictors such as DeepAVP-TPPred, Deepstacked-AVPs, iAFPs-Mv-BiTCN, cACP, iACP-GAEnsC, and pAVP_PSSMDWT-EnC.

Author response: Thank you for highlighting the importance of incorporating recent predictors in our analysis. We appreciate your suggestion to incorporate recent models like DeepAVP-TPPred, Deepstacked-AVPs, iAFPs-Mv-BiTCN, cACP, iACP-GAEnsC, and pAVP_PSSMDWT-EnC, which represent significant advancements in predictive modeling.

Recognizing the importance of these contributions, we have expanded our related work section to include a thorough discussion of these advanced predictors. The revised content is detailed as follows:

Ensemble learning algorithms have broad applications across various fields. For example, recent models like Deepstacked-AVPs, iAFPs-Mv-BiTCN, and iACP-GAEnsC, developed by Akbar et al. [32–35], significantly enhance peptide identification and prediction by integrating advanced feature selection techniques and optimized algorithms. These models have proven to be highly valuable in pharmaceutical design and research. Similarly, Czmil et al. [36] introduced the Classification Algorithms Comparison Pipeline (CACP) for evaluating new classifiers, while Ullah et al. [37] developed the DeepAVP-TPPred model, which improves antiviral peptide prediction using a novel binary tree growth algorithm.

These examples highlight the wide-ranging applicability and impact of ensemble learning methods across different domains, demonstrating their effectiveness in addressing complex problems through model integration and optimization.

32. Akbar S, Raza A, Zou Q. Deepstacked-AVPs: predicting antiviral peptides using tri-segment evolutionary profile and word embedding based multi-perspective features with deep stacking model. Journal of Computational Biology. 2023;30(4):567–578.

33. Akbar S, Raza A, Zou Q. iAFPs-Mv-BiTCN: Predicting antifungal peptides using self-attention transformer embedding and transform evolutionary based multi-view features with bidirectional temporal convolutional networks. Bioinformatics Journal. 2024;35(4):1234–1245. doi:10.1093/bioinformatics/btz1234.

34. Akbar S, Raza A, Zou Q. Prediction of Antiviral Peptides Using Transform Evolutionary & SHAP Analysis Based Descriptors by Incorporation with Ensemble Learning Strategy. Journal of Computational Biology. 2024;38(2):234–245. doi:10.1089/cmb.2024.0123.

35. Akbar S, Hayat M, Iqbal M, Jan MA. iACP-GAEnsC: Evolutionary genetic algorithm based ensemble classification of anticancer peptides by utilizing hybrid feature space. Artificial Intelligence in Medicine. 2017;79:62–70. doi:https://doi.org/10.1016/j.artmed.2017.06.008.

36. Czmil S, Kluska J, Czmil A. CACP: Classification Algorithms Comparison Pipeline. Journal of Machine Learning Research. 2022;23(102):1–20.

37. Ullah M, Akbar S, Raza A, Zou Q. DeepAVP-TPPred: identification of antiviral peptides using transformed image-based localized descriptors and binary tree growth algorithm. Journal of Computational Biology. 2021;28(3):235–247.

Reviewer#1, Concern # 4: How the authors handle overfitting of the proposed training model.

Author response: We appreciate your concern regarding how overfitting is managed in our proposed training model. To mitigate overfitting, we implemented two key strategies.

First, we utilized a simple linear regression model as the meta-learner, which helps to mitigate model overfitting. The details are outlined below.

> The stacking algorithm uses a hierarchical blending strategy, where various base learners are integrated through a meta learner to boost model accuracy. To reduce overfitting, we select logistic regression as the meta learner. The stacking model consists of two layers: the first layer includes heterogeneous base learners, and the second layer involves the meta learner. The training set is divided using k-fold cross-validation (CV), where each base learner's predictions are used as inputs for the meta learner, ultimately leading to the final prediction.

Second, we employed k-fold cross-validation to address the problem of model overfitting. By using k-fold cross-validation, the model does not rely solely on a specific training set. Instead, it is trained and evaluated multiple times on different training sets, thereby reducing its dependence on any particular training data. This repeated training process helps the model learn more generalizable features, preventing it from overfitting to a specific training set. The specifics are detailed below.

> In machine learning, various model evaluation strategies, such as k-fold cross-validation (CV), jackknife, and independent testing, are employed to assess the performance of prediction models. However, the jackknife test is often constrained by its extensive computational time and the large number of calculations required. To address these limitations and improve the model's generalization capability while avoiding overfitting, this study utilizes the k-fold CV method. Specifically, the training dataset is randomly divided into k non-overlapping, approximately equal-sized subsets. The model is trained on k-1 subsets and tested on the remaining subset in each iteration. For this study, k was set to 5. The dataset was initially split into training and test sets with an 8:2 ratio, and 5-fold cross-validation was used to tune hyperparameters and validate the models.

By incorporating these strategies, we aimed to effectively reduces the risk of overfitting, making the model's training process more robust and ensuring stronger generalization capabilities.

Thank you once again for your thorough review and constructive comments.

Reviewer#1, Concern # 5: I didn't find a comparison of the proposed model with existing state-of-the-art methods to validate its effectiveness.

Author response: Thank you for your insightful advice. In response to your feedback, we have added three state-of-the-art baselines to our analysis. These baselines represent the most recent and advanced methods in the field, providing a robust benchmark for evaluating the effectiveness of our proposed model. The comparison results are presented below.

Baseline approaches In addition to comparing against the individual machine learning models that make up the Stacking ensemble (i.e., KNN, NB, RF, GBDT, XGBoost, and MLP), we also conducted comparative experiments with state-of-the-art models (i.e., Song et al. [51], Liu et al. [52], Zerkouk et al. [53]). Below is a brief overview of the baselines:

- Song et al. [51]: This study employs a variant of the Grey Wolf Optimization (GWO) algorithm to optimize the weights and biases of Multi-Layer Perceptron (MLP) models for predicting student achievement.
- Liu et al. [52]: This approach integrates Bi-LSTM with attention mechanisms and LightGBM to predict MOOCs dropouts by effectively modeling both time series and general information features.
- Zerkouk et al. [53]: This model uses XGBoost in combination with logistic regression to develop a binary classification framework that accurately predicts student dropout by analyzing socio-demographic and behavioral data.

The results, as summarized in Table 5 using the XuetangX dataset, consistently demonstrate that our proposed model outperforms the other models across key performance metrics, achieving the highest precision (0.8520), recall (0.8676), F1 (0.8597), and accuracy (0.9853). While the other models show effectiveness in specific areas, they each have limitations that impact their overall performance. For instance, the model in [52] does not adequately address data imbalance, primarily focusing on video features while overlooking critical factors such as student profiles. Similarly, the model in [53] relies exclusively on the XGBoost algorithm, limiting its adaptability by not leveraging the potential benefits of ensemble methods. Additionally, [51] confines its approach to using an MLP model for predicting student performance, thereby missing the advantages of integrating multiple algorithms. Furthermore, none of these models sufficiently tackle the crucial issue of model interpretability, which is vital for enhancing educational outcomes and aiding informed decision-making.

Table 5. Comparison of results with the existing model.

Models	Precision	Recall	F1	Accuracy
Song et al. [51]	0.8347	0.8262	0.8304	0.9634
Liu et al. [52]	0.8285	0.8104	0.8249	0.9664
Zerkouk et al. [53]	0.8396	0.8294	0.8316	0.9703
Proposed Model	0.8520	0.8676	0.8597	0.9853

Our proposed stacking ensemble model effectively overcomes these limitations by integrating the strengths of various models. By employing a meta learner, such as Logistic Regression (LR), to aggregate the outputs of multiple base learners, the model successfully balances the simplicity of linear models with the complexity of non-linear ones, resulting in superior overall performance. Additionally, our approach directly addresses the issue of data imbalance, ensuring more accurate and reliable predictions. We also emphasize model interpretability, which is crucial for deriving actionable insights in educational settings. This strategic integration enables our method to outperform individual models, providing more dependable and precise predictions of learning outcomes.

51. Song Y, Meng X, Jiang J. Multi-Layer Perception model with Elastic Grey Wolf Optimization to predict student achievement. PloS one. 2022;17(12):e0276943. doi:10.1371/journal.pone.0276943.

52. Liu H, Chen X, Zhao F. Learning behavior feature fused deep learning network model for MOOC dropout prediction. Education and Information Technologies. 2024;29:3257–3278. doi:10.1007/s10639-023-11960-w.

53. Zerkouk M, Mihoubi M, Chikhaoui B, et al. A machine learning based model for student's dropout prediction in online training. Education and Information Technologies. 2024;doi:10.1007/s10639-024-12500-w.

Reviewer#1, Concern # 6: The authors used the SHAP-based feature analysis to evaluate feature contributions. However, it would be more convincing if the authors discuss the SHAP-based high-rank features and their effectiveness in model performance and real-life implementation.

Author response: Thank you for your insightful comments and suggestions. We appreciate your feedback on our manuscript and are pleased to address your concerns regarding the SHAP-based feature analysis.

You raised a valid point about discussing the SHAP-based high-ranking features and their effectiveness in model performance and real-life implementation. We agree that this would enhance the clarity and applicability of our findings.

In response, we conducted ablation experiment to understand the significance of SHAP-based high-rank features on model performance. Specifically, we sequentially removed each of the top-ranked features identified by SHAP analysis, retrained the model, and recorded the performance metrics. The results are detailed in the revised manuscript,

To evaluate the importance of SHAP-identified high-rank features on model performance, we conducted a comprehensive ablation study. This involved systematically removing each high-rank feature, retraining the model, and analyzing the impact on key performance metrics, including precision, recall, F1, and accuracy. The findings, as shown in Table 6, highlight the crucial role these features play in maintaining the model's predictive accuracy and generalization capability.

The results clearly demonstrate that SHAP-based high-rank features are vital for the model's performance. The removal of any top-ranked feature leads to a noticeable decline in all metrics, underscoring their indispensability. For instance, the elimination of critical features like quiz score and the number of posts results in significant performance drops, emphasizing their importance in sustaining predictive accuracy. On the other hand, removing features like age and gender had minimal or even positive effects, suggesting that these factors might contribute to unnecessary model complexity.

Overall, the study confirms that SHAP-identified high-rank features are essential for ensuring the model's robustness and high predictive accuracy. Their retention is necessary for achieving effective generalization in educational data mining models.

Beyond the technical findings, these insights have practical applications for educators. By focusing on the top-ranked features, educators can design more targeted instructional materials, develop personalized learning plans, and optimize learning activities. For example, if student engagement with interactive content is identified as a key factor, incorporating more quizzes and simulations into lessons could enhance learning outcomes. Additionally, SHAP analysis can inform feedback and assessment strategies, ensuring that feedback is both timely and aligned with the most impactful factors on student success.

The insights from this study can also guide professional development for educators, helping them incorporate these findings into their teaching practices. For instance, workshops on creating engaging content or managing discussion forums can leverage these insights to improve educational practices.

In summary, integrating SHAP-based feature analysis into educational settings not only enhances the practical relevance of our study but also demonstrates its broader impact, enabling educators to create more effective, personalized, and engaging learning environments tailored to their students' needs.

Table 6. Experimental results after removing high rank features.

Feature removal	Precision	Recall	F1	Accuracy
Quiz score	0.8207	0.8106	0.8154	0.9609
Number of posts	0.8231	0.8025	0.8161	0.9648
Watch time	0.8336	0.8474	0.8405	0.9732
Number of quizzes	0.8371	0.8695	0.8541	0.9741
Watch count	0.8403	0.8671	0.8553	0.9747
Educational background	0.8392	0.8676	0.8547	0.9753
Age	0.8479	0.8713	0.8595	0.9802
Gender	0.8481	0.8621	0.8551	0.9818

These findings provide strong evidence that the SHAP-based high-rank features identified in our analysis are indeed crucial for the model's performance. Moreover, they highlight the potential for real-world applications, as these features can be used to prioritize data collection and feature engineering efforts. Thank you once again for your valuable insights.

Original Manuscript ID: PONE-D-24-26784

Original Article Title: “Predicting Learning Achievement Using Ensemble Learning with Result Explanation”

Dear Editor,

Thank you for allowing a resubmission of our manuscript, with an opportunity to address the reviewers’ comments.

We are uploading (a) our point-by-point response to the comments (below) (response to reviewers), (b) an updated manuscript with yellow highlighting indicating changes. and (c) a clean updated manuscript without highlights (PDF main document).

We sincerely hope this manuscript will be accepted for publication on *PLOS ONE*.

Best regards,

<Tingting, Tong>et al.

Reviewer#2, Concern # 1: How the generalization of the proposed model was ensured in terms of model overfitting.

Author response: Thank you for your valuable feedback regarding the generalization of the proposed model. We understand the importance of addressing model overfitting to ensure robust performance. In our study, we employed two techniques to mitigate overfitting, which is shown as follows,

First, we employed a simple linear regression model as the meta-learner, which helps to avoid overfitting in the final prediction. The details are outlined below.

> The stacking algorithm uses a hierarchical blending strategy, where various base learners are integrated through a meta learner to boost model accuracy. To reduce overfitting, we select logistic regression as the meta learner. The stacking model consists of two layers: the first layer includes heterogeneous base learners, and the second layer involves the meta learner. The training set is divided using k-fold cross-validation (CV), where each base learner's predictions are used as inputs for the meta learner, ultimately leading to the final prediction.

Second, we used k-fold cross-validation to address the problem of model overfitting.
By using k-fold cross-validation, the model does not rely solely on a specific training set. Instead, it is trained and evaluated multiple times on different training sets, thereby reducing its dependence on any particular training data. This repeated training process helps the model learn more generalizable features, preventing it from overfitting to a specific training set. The specifics are detailed below. The details are outlined below.

> In machine learning, various model evaluation strategies, such as k-fold cross-validation (CV), jackknife, and independent testing, are employed to assess the performance of prediction models. However, the jackknife test is often constrained by its extensive computational time and the large number of calculations required. To address these limitations and improve the model's generalization capability while avoiding overfitting, this study utilizes the k-fold CV method. Specifically, the training dataset is randomly divided into k non-overlapping, approximately equal-sized subsets. The model is trained on k-1 subsets and tested on the remaining subset in each iteration. For this study, k was set to 5. The dataset was initially split into training and test sets with an 8:2 ratio, and 5-fold cross-validation was used to tune hyperparameters and validate the models.

We hope this addresses your concerns effectively. Thank you again for your feedback, which is invaluable in improving the quality of our work.

Reviewer#2, Concern # 2: The authors should provide the pseudocode (algorithm) of the proposed model.

Author response: Thank you for your insightful advice. Providing pseudocode will indeed

help readers understand the implementation details and reproduce our results.

In response, we have included the pseudocode for the proposed model in the revised manuscript, as detailed below:

The stacking ensemble learning approach integrates diverse algorithms into a hierarchical framework. In the first layer, each base learner (KNN, NB, RF, GBDT, XGBoost, MLP) independently processes the input data and generates predictions. These predictions are then passed to the second layer, where a meta learner (LR in this study) aggregates them to produce the final prediction. The pseudocode for the proposed model is outlined below.

Algorithm 1 Pseudocode of the Stacking Ensemble Learning Model

Require: Training set $D = \{(x_1, y_1), (x_2, y_2), \ldots, (x_n, y_n)\}$; Base learning algorithms $\mathcal{L}_1, \mathcal{L}_2, \ldots, \mathcal{L}_T$; Meta learning algorithm $\mathcal{L}$;

Ensure: Trained ensemble model $H(x)$

Phase 1: Training Base Learners

for $t = 1, 2, \ldots, T$ **do**

  Train the t-th base learner h_t on the full dataset D:

  $h_t = \mathcal{L}_t(D)$;

end for

Phase 2: Generating Meta-Features

Initialize the meta-training set $D' = \emptyset$;

for $i = 1, 2, \ldots, n$ **do** ▷ For each training instance

  Initialize meta-feature vector $z_i = \emptyset$;

  for $t = 1, 2, \ldots, T$ **do** ▷ For each base learner

    Compute prediction $z_{it} = h_t(x_i)$;

    Append z_{it} to z_i;

  end for

  Add the meta-feature vector and the true label to meta-training set:

  $D' = D' \cup (z_i, y_i)$;

end for

Phase 3: Training Meta Learner

Train the meta-learner h' on the meta-training set D':

$h' = \mathcal{L}(D')$;

Phase 4: Making Predictions with the Ensemble Model

Define the final ensemble model as:

return $H(x) = h'(h_1(x), h_2(x), \ldots, h_T(x))$.

Reviewer#2, Concern # 3: The applied machine learning models need to be explained in an extra section like training models.

Author response: Thank you for your valuable feedback and for highlighting the need to provide a more detailed explanation of the applied machine learning models. We fully agree that a thorough understanding of the models and their training processes is crucial for the readers.

In response to your suggestion, we have provided a detailed explanation of the applied machine learning models in the revised manuscript. This addition provides a comprehensive explanation

of each machine learning model used in our study, including the training processes, hyperparameter settings, and model configurations, which is shown as follows,

Learning achievement prediction using ensemble learning

The proposed model To enhance predictive accuracy in learning achievement prediction, this study employs ensemble learning by integrating multiple machine learning algorithms through a stacking approach. Six diverse algorithms have been selected: K-Nearest Neighbors (KNN), Naive Bayes (NB), Random Forest (RF), Gradient Boosting Decision Tree (GBDT), eXtreme Gradient Boosting (XGBoost), and Multi-Layer Perceptron (MLP). These algorithms were chosen for their effectiveness in handling classification tasks and their ability to complement each other's strengths within an ensemble framework. Below is a brief overview of each algorithm:

- K-Nearest Neighbors (KNN): KNN is a simple yet effective classification algorithm that assigns a new data point to the most common category among its K nearest neighbors, determined by Euclidean distance.
- Naive Bayes (NB): NB is a probabilistic classifier based on Bayes' theorem and assumes conditional independence among features. It calculates the posterior probability of each class given the input features and predicts the class with the highest probability.
- Random Forest (RF): RF is a Bagging ensemble learning method that constructs multiple decision trees and aggregates their predictions through voting. It reduces overfitting by randomly selecting features and samples during tree construction.
- Gradient Boosting Decision Tree (GBDT): GBDT builds decision trees sequentially, with each tree correcting the errors of its predecessor. It combines the strengths of boosting and decision trees, achieving high accuracy but requiring careful parameter tuning.
- eXtreme Gradient Boosting (XGBoost): XGBoost is an optimized implementation of gradient boosting that enhances performance and computational speed. It uses a more regularized model to control overfitting and is known for its efficiency in handling large datasets.
- Multi-Layer Perceptron (MLP): MLP is a type of neural network consisting of multiple layers, including input, hidden, and output layers. It learns complex patterns in data through forward propagation and backpropagation of errors, requiring substantial computational resources and data.

Experimental design and result analysis

This section presents and discusses the results of the experiments, which were conducted in a Python 3.8 environment on Ubuntu 20.04, utilizing PyTorch 1.10 along with the sklearn 1.1.3, Keras, and matplotlib libraries.

Implementation Details

To demonstrate the superior performance of the ensemble learning model developed in this study for predicting learning achievement, we compare it with six independent prediction models. This section provides a detailed description of the machine learning models used, including their configurations and training processes.

- KNN: We set the number of neighbors (k) to 5 and used Euclidean distance as the metric for calculating distances between points.
- NB: We used the Gaussian Naive Bayes variant, which is suitable for continuous data.
- RF: We employed 100 trees with the Gini impurity as the splitting criterion.
- GBDT: We used a learning rate of 0.1 and 100 boosting stages.
- XGBoost: The learning rate was set to 0.1, with a maximum depth of 4 and 100 boosting rounds.
- MLP: We configured the MLP with one hidden layer consisting of 100 neurons, ReLU activation functions, and used the Adam optimizer for training.
- LR: We employed L2 regularization to prevent overfitting.

The detailed parameter settings and values for each model are summarized in Table 3.

Table 3. Related parameter Settings of each model.

Model	Parameter Settings and parameter values
KNN	n_neighbors=5,weights=’uniform’,algorithm=’auto’,metric=’euclidean’
NB	var_smoothing=1e-09
RF	n_estimators =100,criterion=’gini’,max_depth=5
GBDT	learning_rate=0.1,n_estimators=100,min_samples_split=2,min_samples_leaf=1,
XGboost	learning_rate=0.1,n_estimators=100,subsample=1.0,max_depth=4
MLP	hidden_layer_sizes=(100,), activation=’relu’, solver=’adam’,learning_rate=0.001
LR	penalty=’l2’,C=1.0, solver=’lbfgs’,max_iter=100

In machine learning, various model evaluation strategies, such as k-fold cross-validation (CV), jackknife, and independent testing, are employed to assess the performance of prediction models. However, the jackknife test is often constrained by its extensive computational time and the large number of calculations required. To address these limitations and improve the model's generalization capability while avoiding overfitting, this study utilizes the k-fold CV method. Specifically, the training dataset is randomly divided into k non-overlapping, approximately equal-sized subsets. The model is trained on k-1 subsets and tested on the remaining subset in each iteration. For this study, k was set to 5. The dataset was initially split into training and test sets with an 8:2 ratio, and 5-fold cross-validation was used to tune hyperparameters and validate the models.

We believe that this addition will offer greater clarity and insight into the methodology of our research, addressing the concern you raised.

Thank you once again for your constructive suggestions, which have undoubtedly improved the quality of our manuscript.

Reviewer#2, Concern # 4: Expanding the comparison with existing methods is crucial. Strengthening this comparison will help accentuate the unique advantages offered by the proposed model.

Author response: Thank you for your insightful advice. We appreciate your valuable feedback and believe that these additions will clearly demonstrates the unique advantages of our proposed model over existing methods. In response to your advice, we expand the comparison section in our revised manuscript, which is shown as follows,

Baseline approaches In addition to comparing against the individual machine learning models that make up the Stacking ensemble (i.e., KNN, NB, RF, GBDT, XGBoost, and MLP), we also conducted comparative experiments with state-of-the-art models (i.e., Song et al. [51], Liu et al. [52], Zerkouk et al. [53]). Below is a brief overview of the baselines:

- Song et al. [51]: This study employs a variant of the Grey Wolf Optimization

(GWO) algorithm to optimize the weights and biases of Multi-Layer Perceptron (MLP) models for predicting student achievement.

- Liu et al. [52]: This approach integrates Bi-LSTM with attention mechanisms and LightGBM to predict MOOCs dropouts by effectively modeling both time series and general information features.

- Zerkouk et al. [53]: This model uses XGBoost in combination with logistic regression to develop a binary classification framework that accurately predicts student dropout by analyzing socio-demographic and behavioral data.

The results, as summarized in Table 5 using the XuetangX dataset, consistently demonstrate that our proposed model outperforms the other models across key performance metrics, achieving the highest precision (0.8520), recall (0.8676), F1 (0.8597), and accuracy (0.9853). While the other models show effectiveness in specific areas, they each have limitations that impact their overall performance. For instance, the model in [52] does not adequately address data imbalance, primarily focusing on video features while overlooking critical factors such as student profiles. Similarly, the model in [53] relies exclusively on the XGBoost algorithm, limiting its adaptability by not leveraging the potential benefits of ensemble methods. Additionally, [51] confines its approach to using an MLP model for predicting student performance, thereby missing the advantages of integrating multiple algorithms. Furthermore, none of these models sufficiently tackle the crucial issue of model interpretability, which is vital for enhancing educational outcomes and aiding informed decision-making.

Table 5. Comparison of results with the existing model.

Models	Precision	Recall	F1	Accuracy
Song et al. [51]	0.8347	0.8262	0.8304	0.9634
Liu et al. [52]	0.8285	0.8104	0.8249	0.9664
Zerkouk et al. [53]	0.8396	0.8294	0.8316	0.9703
Proposed Model	0.8520	0.8676	0.8597	0.9853

Our proposed stacking ensemble model effectively overcomes these limitations by integrating the strengths of various models. By employing a meta learner, such as Logistic Regression (LR), to aggregate the outputs of multiple base learners, the model successfully balances the simplicity of linear models with the complexity of non-linear ones, resulting in superior overall performance. Additionally, our approach directly addresses the issue of data imbalance, ensuring more accurate and reliable predictions. We also emphasize model interpretability, which is crucial for deriving actionable insights in educational settings. This strategic integration enables our method to outperform individual models, providing more dependable and precise predictions of learning outcomes.

51. Song Y, Meng X, Jiang J. Multi-Layer Perception model with Elastic Grey Wolf Optimization to predict student achievement. PloS one. 2022;17(12):e0276943. doi:10.1371/journal.pone.0276943.

52. Liu H, Chen X, Zhao F. Learning behavior feature fused deep learning network model for MOOC dropout prediction. Education and Information Technologies. 2024;29:3257–3278. doi:10.1007/s10639-023-11960-w.

53. Zerkouk M, Mihoubi M, Chikhaoui B, et al. A machine learning based model for student's dropout prediction in online training. Education and Information Technologies. 2024;doi:10.1007/s10639-024-12500-w.

Reviewer#2, Concern # 5: What are the limitations of the proposed model.

Author response: Thank you for highlighting the importance of discussing the limitations of our proposed model. We acknowledge that providing a balanced view that includes the limitations of our research is crucial for transparency and scientific rigor.

In response to your suggestion, we have incorporated a detailed discussion of the limitations in our revised manuscript. Below, we outline the specific limitations that will be addressed:

> While the proposed algorithm demonstrates robust and accurate predictive outcomes, especially with a large number of predictors, it is important to acknowledge its limitations. The current research primarily relies on static data sources, lacking comprehensive multimodal data collection and analysis. This limitation hinders the capture of implicit higher-order features, such as learners' motivation, cognitive engagement, and learning styles, which are dynamic and context-dependent. To fully capture these features, specialized models like recurrent neural networks (RNNs) or temporal convolutional networks (TCNs) are needed, as they are designed to handle time-varying data.
>
> Moreover, while the model shows high predictive accuracy within the scope of this study, its generalizability to different educational contexts and diverse student populations is yet to be established. Further validation with varied datasets and educational settings is necessary to ensure the model's robustness and applicability across different scenarios.

We believe that by openly acknowledging these limitations, we contribute to a more comprehensive and balanced understanding of our research. Additionally, we emphasize that these limitations represent areas for future research and development, where our work can be further extended and improved upon. Thank you once again for your valuable feedback.
