# Supplementary material for: Predicting learning achievement using ensemble learning with result explanation
Source: PLoS One. 2025 Jan 2;20(1):e0312124. doi: 10.1371/journal.pone.0312124 (PMC11694977; doi:10.1371/journal.pone.0312124)
Supplement: Attachment — Submitted filename: Response to Reviewers.pdf [file pone.0312124.s002.pdf]

Manuscript Revision ID: PONE-D-24-26784R1

Original Article Title: “Predicting Learning Achievement Using Ensemble Learning with Result Explanation”

Dear Editor,

Thank you for allowing a resubmission of our manuscript, with an opportunity to address the reviewers’ comments.

We are uploading (a) our point-by-point response to the comments (below) (response to reviewers), (b) an updated manuscript with yellow highlighting indicating changes. and (c) a clean updated manuscript without highlights (PDF main document).

We sincerely hope this manuscript will be accepted for publication on *PLOS ONE*.

Best regards,

<Tingting, Tong>et al.

Reviewer#1, Concern # 1: In the revised version, the references (32-37) are incorrectly cited. I suggest authors to carefully address such issue according to the following doi links:
Deepstacked-AVPs: doi.org/10.1186/s12859-024-05726-5
iAFPs-Mv-BiTCN: doi.org/10.1016/j.artmed.2024.102860
Prediction of Antiviral Peptides Using Transform Evolutionary & SHAP Analysis Based Descriptors by Incorporation with Ensemble Learning Strategy: doi.org/10.1016/j.chemolab.2022.104682
iACP-GAEnsC: doi.org/10.1016/j.artmed.2017.06.008
CACP: doi.org/10.1016/j.chemolab.2019.103912
DeepAVP-TPPred: doi.org/10.1093/bioinformatics/btae305

Author response: We greatly appreciate the time and effort you have invested in reviewing our manuscript, and we are thankful for your valuable comments and suggestions.

We sincerely apologize for the incorrect DOIs cited in the revised version of our manuscript. After carefully reviewing the references, we have corrected the DOIs in line with your recommendations. The references now accurately reflect the correct citations, with the updated DOI links as follows:

32. Akbar S, Raza A, Zou Q. Deepstacked-AVPs: predicting antiviral peptides using tri-segment evolutionary profile and word embedding based multi-perspective features with deep stacking model. BMC Bioinformatics. 2024;25(1):102. doi:doi.org/10.1186/S12859-024-05726-5.
33. Akbar S, Zou Q, Raza A, Alarfaj FK. iAFPs-Mv-BiTCN: Predicting antifungal peptides using self-attention transformer embedding and transform evolutionary based multi-view features with bidirectional temporal convolutional networks. Artificial Intelligence in Medicine. 2024;151:102860. doi:doi.org/10.1016/j.artmed.2024.102860.
34. Akbar S, Ali F, Hayat M, Ahmad A, Khan S, Gul S. Prediction of Antiviral peptides using transform evolutionary & SHAP analysis based descriptors by incorporation with ensemble learning strategy. Chemometrics and Intelligent Laboratory Systems. 2022;230:104682. doi:doi.org/10.1016/j.chemolab.2022.104682.
35. Akbar S, Hayat M, Iqbal M, Jan MA. iACP-GAEnsC: Evolutionary genetic algorithm based ensemble classification of anticancer peptides by utilizing hybrid feature space. Artificial Intelligence in Medicine. 2017;79:62–70. doi:doi.org/10.1016/j.artmed.2017.06.008.
36. Akbar S, Rahman AU, Hayat M, Sohail M. cACP: Classifying anticancer peptides using discriminative intelligent model via Chou's 5-step rules and general pseudo components. Chemometrics and Intelligent Laboratory Systems. 2020;196:103912. doi:doi.org/10.1016/j.chemolab.2019.103912.
37. Ullah M, Akbar S, Raza A, Zou Q. DeepAVP-TPPred: identification of antiviral peptides using transformed image-based localized descriptors and binary tree growth algorithm. Bioinformatics. 2024;40(5):btae305. doi:doi.org/10.1093/bioinformatics/btae305.

Additionally, we have conducted a thorough review of all other references in the manuscript. Upon careful examination, we have ensured that all citations are correctly formatted and

accurately reflect the sources. We deeply regret the oversight in the manuscript and have taken extra care to avoid such errors moving forward.

We sincerely appreciate your attention to detail and your guidance, which has greatly improved the accuracy and quality of our manuscript. We look forward to your further assessment and hope that our revisions meet your expectations.

We apologize once again for the oversight in our previous submission, and we greatly appreciate your careful attention in pointing out this issue.

Thank you once more for your valuable feedback and guidance.
